# The Composition Optimization of Curcumin-Loaded Double Oil–Water–Oil Emulsions and Their Stability Evaluation

**DOI:** 10.3390/molecules29174035

**Published:** 2024-08-26

**Authors:** Kristýna Opustilová, Barbora Lapčíková, Karolina Kocourková, Lubomír Lapčík

**Affiliations:** Faculty of Technology, Department of Food Technology, Tomas Bata University in Zlin, Nam. T. G. Masaryka 5555, 760 01 Zlin, Czech Republic; k1_ondrouskova@utb.cz (K.O.); kkocourkova@utb.cz (K.K.)

**Keywords:** multiple emulsion, curcumin, encapsulation, emulsion stability

## Abstract

This study aimed to optimize the preparation of multiple oil–water–oil (O/W/O) emulsions using varying amounts of Tween 20 emulsifier, different homogenization methods, and optimal preparation temperatures as carriers for encapsulated curcumin. Following the optimization process, the optimal preparation temperature was found to be 25 °C, with a homogenization speed of 10,000 RPM and an emulsifier concentration of 0.5% Tween 20. Subsequently, the effects of physicochemical and viscoelastic properties on the different types of oils used in the outer phase, as well as the impact of storage time, were monitored. The novelty of this work lies in its comprehensive analysis of the stability and encapsulation efficiency of multiple emulsions using various oils, an area that has not been extensively explored before. After identifying the optimal preparation procedure, all samples with different edible oils demonstrated excellent stability and encapsulation efficiency, showing minimal variation in results. The most stable multiple emulsion was found to be the one with coconut oil in the outer phase, exhibiting half the particle size compared to other samples and the lowest encapsulation efficiency losses over 50 days of storage. This study provides new insights into the formulation of stable multiple emulsions for the effective delivery of curcumin and similar bioactive compounds.

## 1. Introduction

Curcumin (1,7-bis-(4-hydroxy-3-methoxyphenyl)-hepta-1,6-diene-3,5-dione), a lipophilic polyphenol derived from turmeric (Curcuma longa), finds widespread use in food, cosmetics, and pharmaceuticals due to its diverse physiological benefits [1], including anti-inflammatory [2], antimicrobial [3], anticancer [4], and antioxidant properties [5]. It also has other effects such as the inhibition of adipocyte differentiation and the promotion of antioxidant activities. Through these diverse mechanisms, curcumin reduces obesity and curtails the adverse health effects of obesity [6] and can also bind with certain heavy metals, such as cadmium and lead, thereby reducing their toxicity [6]. In addition to curcumin, two other curcuminoids occur in lesser amounts in turmeric, namely, demethoxycurcumin and bis-demethoxycurcumin [7,8]. Kim et al. (2017) list cyclocurcumin as another curcuminoid in their publication [9]. However, its practical utility is hampered by challenges such as poor solubility in water, chemical instability, susceptibility to oxidative degradation, and low oral bioavailability [10]. Developing effective delivery systems to overcome these obstacles presents a complex task. Although hydrophobic bioactive compounds are typically unsuitable for direct use in food and beverages, they can be incorporated into formulations to enhance handling, dispersion in water, chemical stability, and effectiveness [11]. Double emulsions offer an optimal delivery strategy that simultaneously encapsulates hydrophilic and hydrophobic bioactive compounds [12]. 

Double emulsions have garnered significant interest in the food industry in recent years and have found application in the production of diverse food items. They are complex, polydisperse systems where both W/O and O/W emulsions coexist simultaneously within the same system. Multiple emulsions are described as “emulsions within emulsions”, as the droplets of the dispersed phase contain even smaller dispersed droplets [13,14]. They are increasingly recognized for their ability to lower calorie content and substitute milk fat in products such as creams, milk, and mayonnaise [15]. Furthermore, they serve as effective carriers for delivering flavors and essential nutrients for nourishing the body [16,17]. Double emulsions may further protect and encapsulate sensitive molecules and bioactive components, thereby gradually releasing them during consumption or digestion [18,19]. In addition, O/W/O double emulsions enable the removal of toxic or unhealthy substances by acting as internal reservoirs to restore substances converted from the external continuous phase into the internal restricted confined phase [16]. However, O/W/O double emulsions are less common, and optimizing their formulation is challenging due to their complex internal structure and inherent thermodynamic instability [16,17] The physicochemical parameters of double emulsions, such as size, stability, rheology, and microstructure of the particles, depend on the nature of the components added to the emulsion system. They are also reliant on processing and storage conditions [16,20]. Elaine (2024) states in her publication that multiple emulsions do not exceed a stability period of two months. The influence of composition, the type of emulsifier used, and preparation temperature have been identified as pivotal factors for the formation of stable emulsions [21].

This article focuses predominantly on the optimization of the method for preparing multiple water-in-oil-in-water (O/W/O) emulsions, encapsulating curcumin within these multiple emulsions, and monitoring their stability over time. The optimization specifically targeted the inner phase of the emulsion and subsequently the resulting multiple emulsion. Emphasis was placed on investigating the quantity of emulsifier, preparation temperature, ratio of oil and aqueous phases, and homogenization method. The stability of the emulsion was monitored using the creaming index, the emulsion stability index, the water freezable index, rheology measurements, and confocal laser microscopy over a period of 50 days.

## 2. Results and Discussion

### 2.1. Results of Design Experiment and ANOVA Statistical Analysis 

All process variables in the design were examined at five levels, marked with codes (−α, −1, 0, +1, +α). These codes represent standardized transformations of the original measurement units of the variables. The process variables were standardized using a simple linear transformation, where the highest value of the variable was assigned a value of +1, and the lowest value was assigned a value of −1. The average of these two values was denoted as 0. The values −α and +α were then used to determine the minimum and maximum values of the variable [22,23,24]. The independent variables corresponding to the levels and codes are presented in Tables S1 and S2 of the Supplementary Materials. The value of the correlation coefficient (R^2^) was 95.36% for IP and 99.76% for ME, indicating an acceptable correlation between the proposed statistical model and the experimental data. The significance of the independent factors was determined using analysis of variance (ANOVA) based on *p*-values, with significance set at *p* < 0.05 [25]. The analysis was performed using Design Expert software, version 13 (Stat-Ease Inc., Minneapolis, MI, USA). To visualize the effect of the water-to-oil phase ratio, optimal temperature, and homogenization method on IP, as well as the oil-to-water phase ratio and the amount of emulsifier on ME, three-dimensional surface plots are provided (Figures S1 and S2 in the Supplementary Materials). 

To achieve accurate values of CPI30 and ESI for the preparation of emulsions with maximum stability, numerical optimization was used. For IP, the optimal temperature was selected as 25 °C, the homogenization speed was set at 25,000 RPM, and the water-to-oil ratio was 9:1. For ME, the optimal water-to-oil ratio was chosen as 2:3 with an optimal emulsifier concentration of 0.5%. From the optimization method, it was found that the stability of emulsions was most significantly influenced by the temperature of emulsion preparation. Even a slight increase in temperature during the manufacturing process led to the rapid separation of the water and oil phases. The homogenization process also had a significant effect on the stability of the emulsions. The higher the rotational speed used during homogenization, the more stable the emulsions were prepared. The speed of rotation during homogenization is related to the size of the droplets formed; the smaller the droplets of the emulsion, the more stable the emulsion is. However, for multiple emulsions, it was not possible to use high rotational speeds, as this could lead to the disruption of the droplets in the internal phase of the emulsion and the destabilization of the emulsion. Zhou et al. (2022) state in their work that stable emulsions can be prepared at a speed of 8000 RPM. It is also not advisable to exceed 14,000 RPM, as at such high speeds, the film of the IP emulsion is already compromised. Another important factor is the amount of emulsifier. If there is no emulsifier in the system, it is not possible to create a stable emulsion for an extended period. As the concentration of the emulsifier in the system increases, the created emulsion becomes more stable [26]. However, the use of emulsifiers in food is limited by the European Parliament and Council Regulation (EC) No. 1333/2008, which states that the maximum allowed amount for Span 80 and Tween 20 is 1000 mg/kg. However, the oil-to-water ratio did not have a significant impact on emulsion stability; only the viscoelastic properties of the prepared emulsions changed. With an increasing content of the oil phase, the emulsion became more liquid, whereas if there was a larger proportion of the water phase, the emulsion became firmer. The selection of the water-to-oil phase ratio thus primarily depends on the application of the specific emulsion and consumer preferences. 

### 2.2. Determination of Emulsion Stability by the Creaming Index (CPI) 

**Figure 1:**
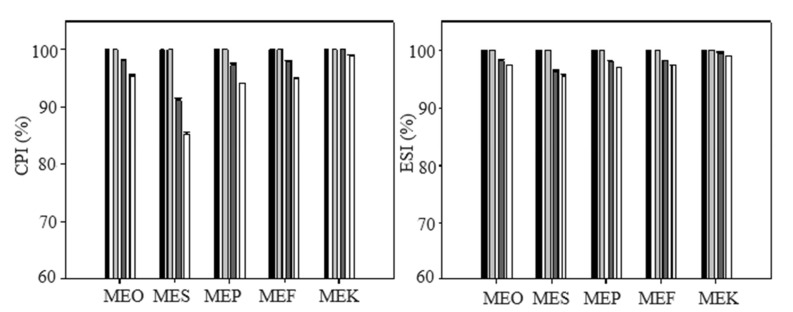
The creaming index (CPI) and emulsion stability index (ESI) of multiple emulsions dependent on the storage time: samples measured immediately after the preparation (black columns), after 15 days (light grey columns), after 30 days (dark grey columns), and after 50 days (white columns). Error bars represent the standard deviation of three measurements. Sample labels: multiple emulsions with olive oil (MEO), sunflower oil (MES), pumpkin seed oil (MEP), flaxseed oil (MEF), and coconut oil (MEK).

The data obtained from the calculation of the creaming index (CPI) (Figure 1 and Figure S1) revealed a remarkable level of stability throughout the experiment. After 15 days of storage, all samples exhibited a CPI value of 100%, indicating that no phase separation occurred during the first 15 days. After 30 days of storage, slight separation began to occur. The highest degree of separation after 30 days was observed in the sample containing sunflower oil (MES), where a CPI value of 91.3 ± 0.3% was measured. Other samples maintained CPI values above 97%. After 50 days of storage, a slight decrease in CPI was observed in all samples, but none of the samples had a CPI value below 85%. The lowest CPI value was again measured in the sunflower oil sample at 85.3 ± 0.2%. Data obtained from the measurement of the emulsion stability index (ESI) were similar to the data obtained from the CPI. After 15 days of storage, all samples reached ESI values of 100%, indicating no signs of separation. After 30 days, the ESI slightly decreased, with the lowest ESI value again detected in the MES sample at 96.25 ± 0.35%. Other samples had ESI values above 98%. After 50 days of storage, the ESI in all samples slightly decreased again, with all samples showing ESI values above 95%, indicating excellent stability. 

### 2.3. Particle Size 

**Figure 2:**
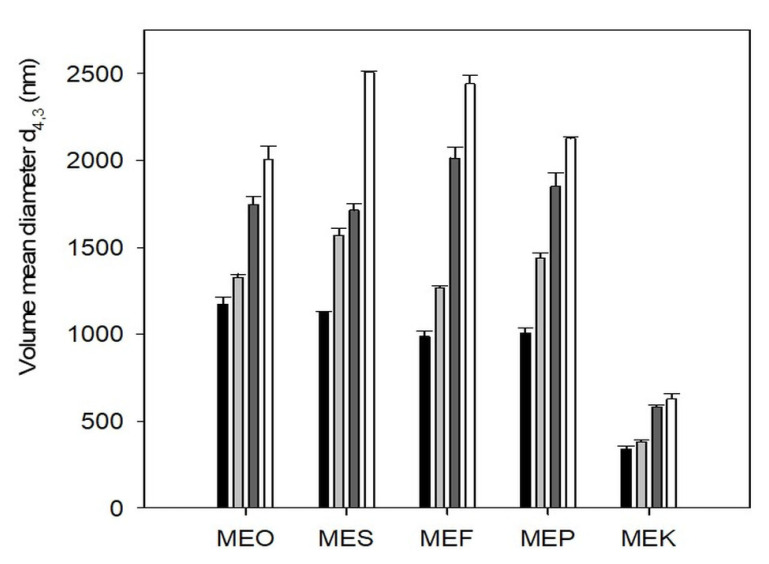
The particle size of multiple emulsions as volume mean diameters d_4,3_ at different storage times: samples measured immediately after the preparation (black columns), after 15 days (light grey columns), after 30 days (dark grey columns), and after 50 days (white columns). Error bars represent the standard deviation of three measurements. Sample labels: multiple emulsions with olive oil (MEO), sunflower oil (MES), flaxseed oil (MEF), pumpkin seed oil (MEP), and coconut oil (MEK).

The particle size was measured using the dynamic light scattering method, and the results are presented in Figure 2. An increasing trend in particle size with storage time was observed for all samples. The largest increase in particle size was recorded for the MES and MEF samples, where the particle size increased by more than 2×. In the MEF sample, the particle size was measured at (986.1 ± 6.4) nm immediately after preparation, and it increased to (2440.4 ± 6.8) nm after 50 days of storage. The MEP sample had an initial particle size of (1005.2 ± 7.5) nm, which increased to (2127.1 ± 5.9) nm after 50 days. In contrast, the smallest particle size was detected in the MEK sample, with an initial measurement of (341.2 ± 5.7) nm immediately after preparation, which increased to (626.9 ± 9.9) nm after 50 days of storage. Similar particle size growth trends were observed in the MEO and MEP samples during storage. The initial value was detected at (1173.4 ± 9.5) nm and (1005.2 ± 7.5) nm, respectively. After 50 days of storage, the particle size increased to (2002.9 ± 7.8) nm for the MEO sample, while the MEP sample showed a larger increase to (2127.1 ± 5.9) nm. Similar particle size values were determined in a study by Zhi et al. (2024), who prepared an emulsion with sunflower oil. In this publication, the values ranged from 1300 to 1900 nm, depending on the amount of emulsifier and the homogenization method used [16]. Dynamic light scattering (DLS) polydispersity patterns were changed from 0.005 to 0.500 during 14 days of storage for MEK, MEO, MEF, and MEP samples. However, the sample MES exhibited an equilibrium polydispersity of 0.250 after storage, which explains the monodisperse-to-polydisperse system change observed after the samples’ storage.

### 2.4. Results of Encapsulation Efficiency 

**Figure 3:**
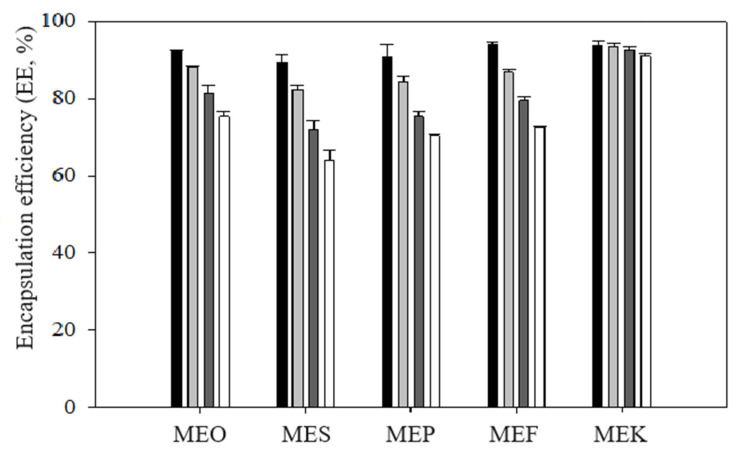
The encapsulation efficiency values of the prepared emulsions during their storage: samples measured immediately after the preparation (black columns), after 15 days (light grey columns), after 30 days (dark light columns), and after 50 days (white columns). Error bars represent the standard deviation of three measurements. Sample labels: multiple emulsions with olive oil (MEO), sunflower oil (MES), pumpkin seed oil (MEP), flaxseed oil (MEF), and coconut oil (MEK).

The results of the encapsulation efficiency (EE) are shown in Figure 3. The results clearly indicate a decrease in EE during storage for most samples. The MEO, MES, MEF, and MEP samples demonstrated a gradual decline in EE with increasing storage time, likely due to the breakdown of the emulsion structure. Remarkably, the MEK sample exhibited a high EE throughout the storage period monitored. Immediately after preparation, the EE for MEK was 93.79 ± 1.03%, and after 50 days of storage, it decreased by less than 3% to (90.96 ± 0.82)%. These data correlated with the findings published by Hsieh et al. (2022), who studied the encapsulation of curcumin into a multiple emulsion with coconut oil and reported an encapsulation efficiency of over 90% throughout the storage period (30 days) [27]. The lowest encapsulation efficiency was observed in the MES sample. Immediately after preparation, the EE was (89.3 ± 2.1)%, and after 50 days of storage, it decreased to (63.88 ± 2.71)%. The MEO, MEF, and MEP samples showed similar encapsulation efficiency, with all samples achieving EE values of over 90% immediately after preparation, followed by a decline in EE during storage. Similar EE values for curcumin were reported by Aditya et al. (2015). The authors investigated EE in multiple emulsions with olive oil and observed an encapsulation efficiency of 88% immediately after preparation. After 50 days of storage, the values decreased to approximately 70% [28]. The data on the encapsulation efficiency of multiple emulsions in different storage times are presented in Table S3 (Supplementary Materials).

The higher encapsulation efficiency observed in the sample containing coconut oil is likely due to the different fatty acid composition present in coconut oil compared to other oils used in this experiment. Unlike other oils, coconut oil predominantly contains saturated fatty acids (95%) with shorter chains, mainly lauric acid (45–53)%, myristic acid (18–21)%, and caprylic acid (8–9)% [29], resulting in a solid consistency at room temperature. This characteristic prevents the migration of droplets from the inner to the outer oil phase, increasing both encapsulation efficiency and the overall emulsion stability [16]. In agreement with this, sunflower oil, which exhibited the lowest EE, contained the least amount of saturated fatty acids, only 6.5%. 

### 2.5. Rheology 

**Figure 4:**
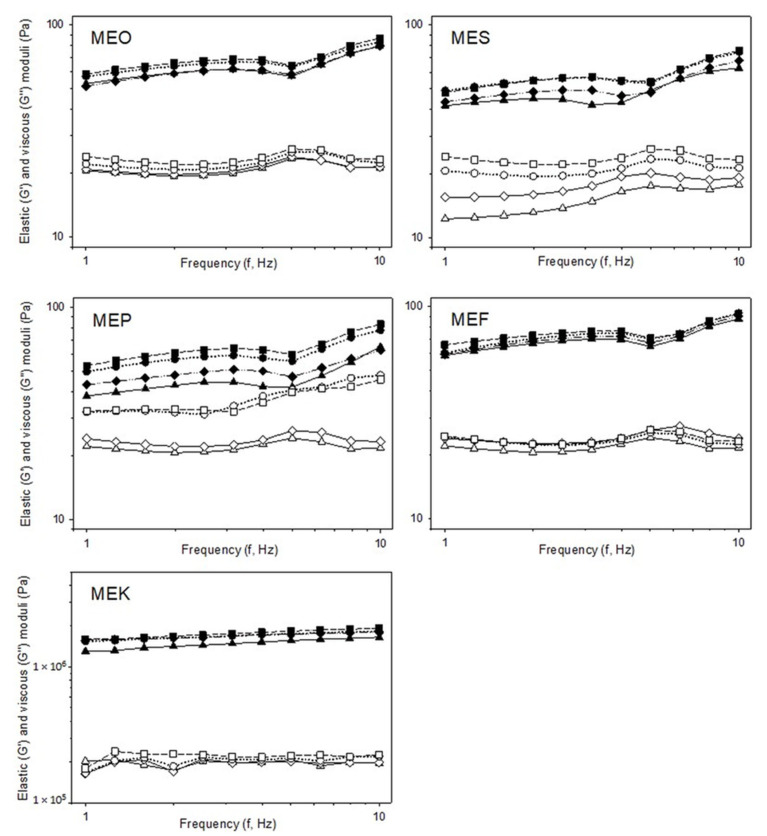
The effect of storage time on viscoelastic properties represented by elastic modulus G′ (closed symbols, Pa) and viscous modulus G″ (open symbols, Pa) of multiple emulsions prepared with different types of edible oil in the outer phase dependent on the frequency in the range of (1.0–10.0) Hz: measurement immediately after the preparation (triangles with full line), after 15 days (diamonds with dash-and-dot line), after 30 days (circles with dotted line), and after 50 days (squares with dashed line). Sample labels: multiple emulsions with olive oil (MEO), sunflower oil (MES), pumpkin seed oil (MEP), flaxseed oil (MEF), and coconut oil (MEK).

Rheological analysis was applied to determine information on the viscoelastic properties and consistency of the samples studied [30]. The development of elastic (G′) and viscous (G″) moduli over time for multiple emulsion samples as a function of frequency (1.0–10.0 Hz) is shown in Figure 4. All tested samples exhibited the predominance of elastic behavior over the viscous one (G′ > G″; *p* < 0.05) in the entire frequency range. According to Saha and Bhattacharya [30], viscoelastic systems exhibiting higher values of the elastic modulus G′ over the viscous modulus G″ are termed gels. The highest elastic modulus G′ was recorded for sample MEK manufactured with coconut oil. This is due to the fatty acid composition of coconut oil and its rigid structure at room temperature [31]. Immediately after preparation, the elastic modulus G′ of the MEK sample was (1599.67 ± 29.15) kPa. The elastic modulus decreased with storage time, reaching a value of (1301.67 ± 38.18) kPa after 50 days of storage. MEO, MES, MEF, and MEP samples showed similar elastic modulus values. The G′ values immediately after preparation were (58.76 ± 3.30) Pa for the MEO sample and (66.09 ± 3.72) Pa for the MEF sample. The MES and MEP samples showed lower values; specifically, in the MES sample, it was (48.18 ± 3.26) Pa, and in the MEP sample, it was (52.91 ± 2.52) Pa. All these values were detected in 1 Hz. The values of G′ and G″ decreased with storage time for all samples, with the smallest difference observed for the MEK sample. According to Pal (2017), changes in viscoelastic properties could be caused by emulsion coalescence associated with the increase in emulsion droplet size or the polydispersity of the emulsion system [32]. This correlates with the data on emulsions’ particle size presented in Section 2.3. For elastic and viscous moduli data, refer to Appendix S3 (Tables S4–S6).

**Figure 5:**
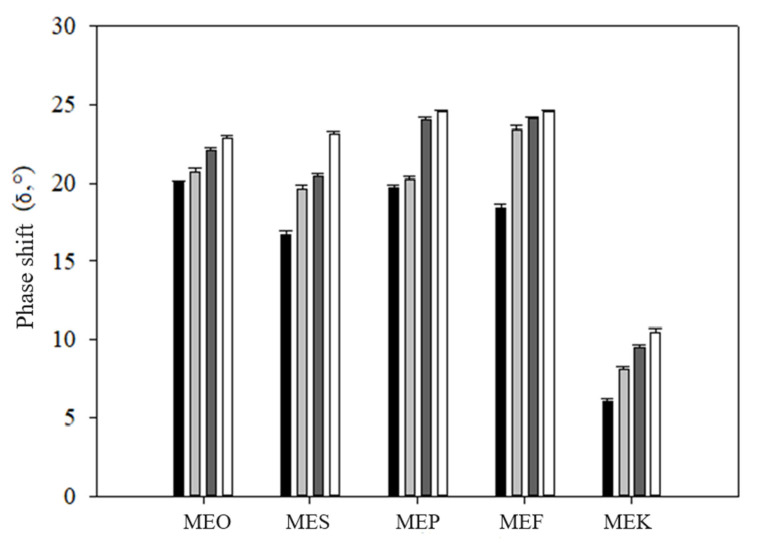
The phase shift (δ, °) of multiple emulsions during the storage time: samples measured immediately after the preparation (black columns), after 15 days (light grey columns), after 30 days (dark grey columns), and after 50 days (white columns). Error bars represent the standard deviation of three measurements. Sample labels: multiple emulsions with olive oil (MEO), sunflower oil (MES), pumpkin seed oil (MEP), flaxseed oil (MEF), and coconut oil (MEK).

The values of phase shift (δ), as presented in Figure 5, also support the conclusion that the stability of the samples was reduced over the storage time observed (*p* < 0.05). From these results, it follows that the strength and elasticity of the samples decreased with storage time, and the emulsion became more fluid. As reported by Janmey et al. (2007), the increase in the phase angle suggests a tendency toward more viscous behavior, whereas the decrease indicates a shift toward elasticity [33]. This correlates with the obtained results. Figure 4 shows that the MEK sample exhibited the lowest values of phase shift, which is due to its stiffest consistency. For all samples, an increase in phase shift over the storage period was observed, indicating the thinning of emulsions over time.

### 2.6. Differential Scanning Calorimetry 

**Figure 6:**
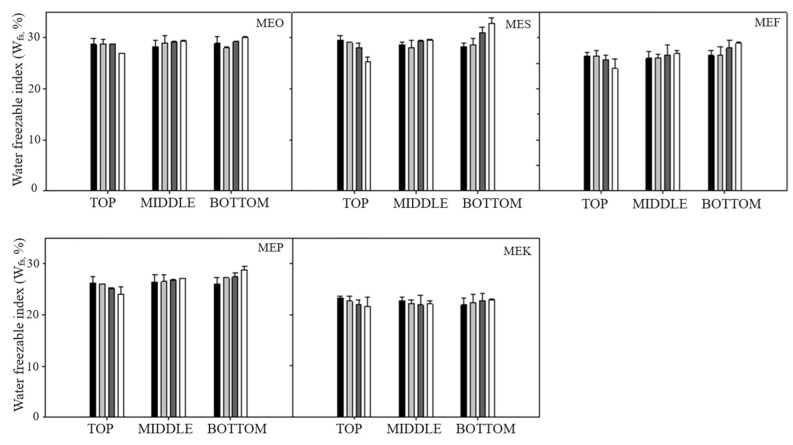
The water freezable index (W_fs_) determined for different parts of the emulsion sample (top, middle, and bottom), as a function of storage time: samples measured immediately after the preparation (black columns), after 15 days (light grey columns), after 30 days (dark grey columns), and after 50 days (white columns). Error bars represent the standard deviation of three measurements. Sample labels: multiple emulsions with olive oil (MEO), sunflower oil (MES), flaxseed oil (MEF), pumpkin seed oil (MEP), and coconut oil (MEK).

The stability of formulated emulsions was evaluated by DSC analysis. Measurements were performed at three distinct positions within the emulsion (top, middle, and bottom) to monitor emulsion destabilization, as can be seen in Figure 6. We observed the emulsion degradation over time due to the coalescence phenomenon, leading to the creaming of emulsion where oil rose to the top and water dropped to the bottom. This phenomenon was confirmed by the data of the creaming index discussed in Section 2.2. Over the monitored storage times, the water freezable index (W_fs_) decreased in the upper region of the emulsion, owing to water dropping at the bottom. Conversely, W_fs_ values exhibited an increase in the bottom of the emulsion. For all the analyzed emulsions, we observed the same trend of W_fs_ values. The smallest differences in all emulsion layers were noticed for the MEK sample, indicating the least degradation of the emulsion. Immediately after preparation, W_fs_ values in the upper layer of the emulsion reached (23.26 ± 0.35)%, which decreased to (21.65 ± 0.82)%; at the bottom, the values were (22.59 ± 1.31)% immediately after preparation and increased to (23.17 ± 0.18)% after 50 days. Similar values were recorded for the MEO sample; however, a significant increase in W_fs_ was observed in the bottom layer of the emulsion. Immediately after preparation, the W_fs_ value was (23.15 ± 1.18)%, which increased to the value (27.89 ± 0.85)% after 50 days. The highest W_fs_ values in the emulsion layers were observed for the MES sample immediately after preparation: (28.71 ± 1.7)% in the top layer, (27.13 ± 1.13)% in the middle layer, and (27.98 ± 1.26)% in the bottom layer. Similar W_fs_ values were observed for MEF and MEP samples, ranging between 26 and 27% immediately after preparation. This can be associated with particle size measurement, where MEF and MEP samples showed the same increase in particle size at the monitored storage time. 

### 2.7. Results of the Confocal Laser Microscopy 

**Figure 7:**
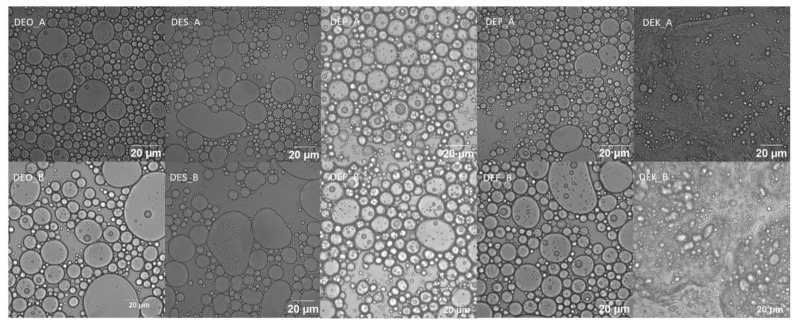
Confocal laser scanning microscopy images of multiple emulsions. Images labeled as A were taken immediately after the emulsions’ preparation, while images labeled as B were taken after 50 days of storage time.

The results of CLSM measurements immediately after preparation and after 50 days of storage are shown in Figure 7. The structure of multiple emulsions in all the prepared samples is confirmed from the images. The CLSM results correlate with particle size measurements, indicating that the particle size in the MEK sample is more than half smaller than the other samples under study. Based on the results, it is also evident that after 50 days of storage, coalescence occurred, leading to an increase in the size of emulsion particles.

## 3. Materials and Methods

### 3.1. Materials

Curcumin (95% total curcuminoid content, Alfa Aesar, Karlsruhe, Germany), Nigella seed oil (100% organic plant oil extracted from Nigella Sativa plant, Nobilis Tilias.r.o, Prague, Czechia), Tween 20 (M = 1.228 g/mol, Sigma-Aldrich, Merck, Burlington, MA, USA), κ-carrageenan (MW = 4.31 × 10^5^ Da, Sigma-Aldrich Ltd.,Burlington, MA, USA), SPAN 80 (M = 460 g, Carl Roth, Karlsruhe, Germany), phosphate buffer (pH 7.0, Ing. Petr Švec—PENTA s.r.o., Prague, Czechia), methanol (Sigma-Aldrich, Ltd., Burlington, MA, USA), and oils (olive, sunflower, pumpkin, flex seed, and coconut) were purchased from the market network. 

#### Design Experiment and ANOVA Statistical Analysis 

The experiments were conducted using a central composite design, where 20 randomized experiments were performed for the inner emulsion phase (IP) and 20 for multiple emulsion (ME). The samples were prepared according to the methods described in Section 3.2.1 and Section 3.2.2, with the modification that sunflower oil was used in both the internal and external phases, and the emulsions did not contain curcumin.

To evaluate the effectiveness of the selected independent factors, namely the ratio of oil-to-water phase (X_1_, 5:95–30:70), preparation temperature (X_2_, 25–70 °C), homogenization process (X_3_, 15,000–25,000 RPM) in the inner phase, the ratio of oil-to-water phase (X_1_, 30:70–70:30), emulsifier amount (X_2_ = 0.1–1 mL), and homogenization process (X_3_, 1000–12,000 RPM) in multiple emulsions, the creaming index after 30 days of storage (CPI_30_) (Y_1_, %) and the emulsion stability index (ESI) (Y_2_, %) were used as experimental responses. The CPI and ESI were determined according to the procedure described in Section 3.3 and Section 3.4. To minimize the pure error, the center point (X_1_ = 17.5:82.5, X_2_ = 47.5 °C, X_3_ = 20,000 RPM) in the inner phase and the center point in multiple emulsions (X_1_ = 50:50, X_2_ = 0.55 mL, X_3_ = 6500 RPM) were repeated five times. A polynomial model of the second order with linear (X_i_), quadratic (X_i2_), and interaction (X_i_X_j_) independent factors was used to investigate the correlation between the CPI_30_ and ESI. After completing the experiments, the quadratic model was adjusted to the response data calculated using Design Expert software (Stat-Ease, version 13.0.5.0). The complete quadratic model for *k* variables contained (*k* + 1)(*k* + 2)/2 parameters, which were determined using Equation (1) as follows:

(1) $$Y_{i}=\beta_{0}+\sum_{i}\beta_{i}X_{i}+\sum_{i}\beta_{ii}X_{i}^{2}+\sum_{i<j}\sum_{j}\beta_{ij}X_{i}X_{j}$$

where *β_i_* represents the coefficients of the linear terms *X_i_*, *β_ii_* represents the coefficients of the quadratic terms *Xi^2^*, and *β_ij_* represents the coefficients of the interaction terms *X_i_X_j_*.

### 3.2. Preparation of Model Samples 

The preparation of multiple emulsions was carried out using a two-step emulsification process. 

#### 3.2.1. Preparation of the Inner Emulsion Phase (O/W) 

The inner oil phase (O_1_) was prepared by dissolving curcumin (95% total curcuminoid content, Alfa Aesar, Karlsruhe, Germany) in Nigella seed oil (100% organic plant oil extracted from Nigella Sativa plant, Nobilis Tilia s.r.o, Prague, Czechia). The oil was stirred on a magnetic stirrer (IKA Rh basic2, Staufen im Breisgau, Germany) at a temperature of (50.0 ± 1.0) °C for at least 1 h for complete dissolution. Subsequently, the aqueous phase (W) was prepared by dissolving κ-carrageenan (Sigma-Aldrich s.r.o., Prague, Czechia); MW = 4.31 × 10^5^ Da) as a stabilizer in distilled water to achieve a 1% solution concentration. The solution was thoroughly mixed and left to hydrate overnight at (4.0 ± 1.0) °C. Tween 20 (M = 1.228 g/mol, Sigma-Aldrich, Merck, Burlington, MA, USA) was added to the carrageenan solution as a stabilizer, and the mixture was stirred on a magnetic stirrer (IKA Rh basic2, Staufen im Breisgau, Germany) for at least 1 h at room temperature. Both solutions were then combined and homogenized using a homogenizer (Silent Crusher M, Heidolph Instruments GmbH & Co. KG, Schwabach, Germany) at 25,000 RPM for 5 min. The composition of the inner phase of the emulsion can be seen in Table 1. 

#### 3.2.2. Preparation of the Multiple Emulsion (O/W/O) 

The multiple emulsion (ME) was created by dispersing the inner emulsion phase from the previous step into the oil phase (O_2_). First, the emulsifier SPAN 80 (M = 460 g, Carl Roth, Karlsruhe, Germany) was dissolved in oil. The solution was stirred for at least 1 h at room temperature on a magnetic stirrer (IKA Rh basic2, Staufen im Breisgau, Germany). The inner emulsion phase was gradually added to the oil phase (O_2_) dropwise. The emulsion was then homogenized at 10,000 RPM for 5 min using a homogenizer (Silent Crusher M, Heidolph Instruments GmbH & Co. KG, Karlsruhe, Germany). A total of five model samples were prepared, each varying in the type of oil used in the outer phase (O_2_)—coconut oil (MEK), flaxseed oil (MEF), olive oil (MEO), sunflower oil (MES), and pumpkin seed oil (MEP). The fatty acid profiles of the oils applied are presented in Table 2. The compositions of individual samples are listed in Table 3. The prepared emulsions were stored at (4.0 ± 1.0) °C for further analysis.

### 3.3. Determination of Emulsion Stability by the Creaming Index (CI) 

The creaming index provides indirect information about the aggregation of droplets and the degree of flocculation of droplets in the system [34]. During storage, there is a phase separation into a cream layer at the top and a serum layer at the bottom of the tube. The emulsion (10 mL) was pipetted into a glass tube (inner diameter 1.5 cm, height 15 cm), sealed, and stored at room temperature. The separation of the cream and serum layers was monitored. The separation of the layers was observed immediately after preparation, as well as at 7 days, 15, 30, and finally 50 days after the pipetting into the tube. The creaming index was calculated using Equation (2) as follows:

(2) $$CI(\%)=\left(1-\frac{HS}{HE}\right)\times 100$$

where *HS* is the height of the serum layer, and *HE* is the total height of the emulsion.

### 3.4. Determination of the Emulsion Stability Index (ESI) 

The emulsion stability index was measured as per the method described by Nikzade et al. (2012), with slight modifications. The emulsions (10 mL) were pipetted into 50 mL polypropylene centrifuge tubes (inner diameter 29.1 mm, height 114.4 mm, conical bottom) and sealed with plastic caps. The samples were then centrifuged at 6000 RPM for 20 min using an EBA 21 centrifuge (Hettich Zentrifugen, Mülheim an der Ruhr, Germany) [35]. The emulsion stability (ESI) was calculated according to Equation (3) as follows:

(3) $$ESI\;(\%)=\left(1-\frac{S_{1}}{S_{2}}\right)\times 100$$

where *S*_1_ is the volume of the separated lower emulsion layer, and *S*_2_ is the total volume of the emulsion. The samples were measured in duplicate immediately after preparation and 50 days after preparation.

### 3.5. Particle Size Measurement 

The particle size was determined by measuring the dynamic light scattering using a ZetaSizer (Brookhaven Instruments, Nashua, NH, USA) at a fixed angle of 90°, a refractive index of 1.3590, and a wavelength of 658 nm. To eliminate the effect of multiple scattering, the samples were diluted 100 times in phosphate buffer (pH 7.0, Ing. Petr Švec—PENTA s.r.o., Prague, Czechia Republic). The scattered light intensity was converted to particle size using the Stokes–Einstein equation. The recorded values were expressed as the volume mean diameter (d_4,3_) of five repetitions. 

### 3.6. Encapsulation Efficiency 

The determination of encapsulation efficiency was performed according to the method published by Prieto et al. (2020), with minor modifications. An aliquot of the emulsion (25.0 ± 0.5) mg was dissolved in 10 mL of methanol (Sigma-Aldrich, Ltd., USA); this solution was gently stirred for 30 s and then filtered through syringe nylon filters (membrane diameter 13 mm, pore size 0.22 µm, Labstore, UK). Subsequently, absorbance was measured using a Shimadzu spectrophotometer (UV mini-1240, Tokyo, Japan) at a wavelength of 420 nm. Quartz cuvettes were used for the measurements (spectral range 200–2500 nm, length 10 mm, volume 3.5 mL, Hellma GmbH & Co. KG, Müllheim, BW, Germany) [36]. The release of curcumin from the emulsion was measured immediately after preparation, and after 15, 30, and 50 days, when each sample was measured three times. The encapsulation efficiency was calculated according to Equation (4) as follows:

(4) $$EE\;\left(\%\right)=\left(1-\frac{A}{B}\right)\times 100$$

where *A* is the total amount of curcumin in the emulsion, and *B* is the extracted curcumin. The concentration of curcumin was derived from the calibration curve: y = 0.149x − 0.0044y (R^2^ = 0.993). All samples were measured at least in three replicates. 

### 3.7. Dynamic Oscillatory Rheology 

To determine the viscoelastic properties of the samples, an oscillator shear rheometer Kinexus (Malvern, UK) with a plate-to-plate geometry (parallel plate geometry diameter was 40 mm, gap 1 mm) at temperature (20.0 ± 0.1) °C was used. In order to determine the linear viscoelastic region, the stress sweep test was performed at 1 Hz in the range of 0.1–20 Pa. The frequency sweep test was run in the range of 0.1–10 Hz in the linear viscoelastic region, and the applied stress was 1.0 Pa for MEO, MES, MEF, and MEP and 20.0 Pa for MEK at 20 °C. Viscoelastic measurements were carried out in an oscillating mode within the region of linear viscoelasticity with a shear stress amplitude value of 1.0 Pa, frequency ranging from 1 to 10 Hz, and a gap of 1 mm. The elastic (G′, Pa) and viscous (G″, Pa) moduli were recorded as a function of frequency f (Hz) using the rSpace software (Malvern Panalytical, version 1.17.2398). Furthermore, the phase shift (δ, °) was determined according to Equation (5) as follows:

(5) $$\delta ={tan}^{-1}G^{\prime \prime }/G^{\prime }$$

A reference frequency of 1 Hz was used for the data presentation. All rheological measurements were repeated at least three times [37].

### 3.8. Determination of Emulsion Stability by Differential Scanning Calorimetry (DSC)

The stability of the emulsions was monitored using a differential scanning calorimeter DSC 250 (TA Instruments, New Castle, DE, USA) following the procedure reported by Dalmazzone et al. (2009) [38]. Briefly, (15.0 ± 0.5) mg of emulsion separated from different emulsion layers (top, middle, and bottom) was weighed into a hermetically sealed pan. The experimental conditions were set as follows: cooling process from 25 °C to −50 °C (at a cooling rate of 10 °C/min), maintaining the isothermal process for 1 min, followed by a heating process from −50 °C to 30 °C (at a heating rate of 5 °C/min). All experiments were conducted in a nitrogen atmosphere (nitrogen flow rate 50 mL/min). An empty pan was used as a reference. We analyzed the melting peak temperature of the water as an onset temperature (T_o_), peak temperature (T_p_), and enthalpy of fusion (Δ*H_e_*, J/g) in emulsion layers [38]. A baseline was subtracted from the measured signal to interpret the measured data. To determine the energy required for the phase transition, the area under the corresponding peak on the thermoanalytical curve was integrated using TA instrument software (TRIOS 5.7.0.56). To determine the stability of the emulsions, the water content (*W_fs_*, %) was calculated using Equation (6) as follows:

(6) $$W_{fs}(\%)=\frac{∆H_{e}}{∆H_{H_{2}O}}\times 100$$

where $∆H_{e}$ is the fusion enthalpy of emulsion, and $∆H_{H_{2}O}$ is the latent heat of pure water (333.5 J/g), as reported by Tylewicz et al. (2016) [39]. Data were analyzed as the average values of three measurements.

### 3.9. Confocal Laser Microscopy 

Samples were characterized with a confocal laser scanning microscope, model FV3000 (Olympus, Tokyo, Japan). A drop of emulsion was deposited on a microscopic slide and covered with a cover slip. The observation of curcumin staining was made with the excitation and emission wavelengths of 428 and 536 nm. Microscopic objectives with a magnification of 10× to 60× with a zoom of 1.5 to 2.5 were used. 

### 3.10. Statistical Analysis 

The obtained data were examined for normal distribution (Shapiro–Wilk test; significance level set at (*p* < 0.05). However, the use of typical parametric tests was rejected because normal distribution was not acceptable for all data (*p* > 0.05). Therefore, the data were analyzed using non-parametric analysis of variance Kruskal–Wallis and Wilcoxon tests with a significance level set at (*p* < 0.05). Statistical methods were applied according to Granato et al. (2014), who evaluated statistics in food science and technology. The data were evaluated using the statistical software SigmaPlot, version 12.5 (Systat Software, San Jose, CA, USA) [40]. 

## 4. Conclusions

In conclusion, this study successfully optimized the preparation process of multiple oil–water–oil (O/W/O) emulsions as carriers for curcumin encapsulation. The optimal conditions identified included a preparation temperature of 25 °C, a homogenization speed of 10,000 RPM, and an emulsifier concentration of 0.5% of Tween 20. Analyses revealed that emulsions containing coconut oil in the outer phase exhibited the highest stability and encapsulation efficiency. In the case of using oils containing unsaturated fatty acids, olive oil was the most suitable for the preparation of multiple emulsions, as indicated by the results of CPI, EE, particle size distribution, CLSM, and W_fs_. In terms of emulsion stability, polyunsaturated fatty acid content appeared to be the most important factor. For MEF and MEP samples, we observed similar results regarding the stability parameters; from the stability point of view, the MES was the less suitable sample. These findings underscore the versatility and encapsulation effectiveness of multiple emulsions, particularly prepared from coconut and olive oil, as delivery systems for encapsulated bioactive compounds. 

## Figures and Tables

**Table 1:** Composition and processing of the inner phase of the emulsion.

Ingredients	Composition ^1^
Curcumin (g)	1
Nigella seed oil (mL)	10
Distilled water (mL)	90
Tween 20 (g)	0.5
κ-carrageenan (g)	1
**Processing Parameters**	
Homogenization speed (RPM) ^2^	25,000
Homogenization time (min)	5
Temperature (°C)	25

^1^ Ingredient amount per 100 mL of emulsion. ^2^ RPM—rotations per minute.

**Table 2:** Content of fatty acids in oils (data declared by the producers).

Oil Type (Producer)	Content of Fatty Acids (g/100 mL)		
	Saturated Fatty Acid	Monounsaturated Fatty Acids	Polyunsaturated Fatty Acids
Olive (100% Bio extra virgin oil, Headquartes esporao, Espana)	13.1	71.8	6.3
Sunflower (Bio virgin oil, Country life, Slovakia)	6.5	85.8 ^a^	
Flaxseed (100% Bio virgin oil, Greenspol, Hungary)	10.0	20.0	70.0
Pumpkin (Bio 100% virgin oil, Helth lins, The Netherlands)	17.0	25.0	50.0
Coconut (Fair trade, Bio virgin oil, Purity Vision, Czechia Republic)	95.0	5.0 ^a^	

^a^ The total content of mono- and polyunsaturated fatty acids.

**Table 3:** Composition and processing of multiple emulsions.

Ingredients	MEO	MES	MEF	MEP	MEK
Inner phase (mL)	40	40	40	40	40
SPAN 80 (g)	0.5	0.5	0.5	0.5	0.5
Oil (mL)	60	60	60	60	60
Oil type	olive	sunflower	flax	pumpkin	coconut
**Processing parameters**					
Homogenization speed (RPM) ^1^	10,000	10,000	10,000	10,000	10,000
Homogenization time (min)	5	5	5	5	5
Temperature (°C)	25	25	25	25	25

^1^ RPM—rotations per minute. Sample labels: multiple emulsions with olive oil (MEO), sunflower oil (MES), flaxseed oil (MEF), pumpkin seed oil (MEP), and coconut oil (MEK).

## Data Availability

The original contributions presented in the study are included in the article and Supplementary Materials; further inquiries can be directed to the corresponding author.

## Supplementary Materials

The following supporting information can be downloaded at: https://www.mdpi.com/article/10.3390/molecules29174035/s1. Table S1: Optimization method for the inner phase of emulsion. Table S2: Optimization method for multiple emulsions. Table S3: Results of encapsulation efficiency of multiple emulsions during the storage period. Figure S1: Response surface plots showing the effect of preparation temperature, oil amount, and homogenization method on creaming index CPI (%) and emulsion stability index ESI (%) for multiple emulsion optimization. Figure S2: Response surface plots showing the effect of emulsifier amount, oil amount, and homogenization method on creaming index (CPI, %) and emulsion stability index (ESI, %) for multiple emulsion optimization. Table S4: Values of elastic moduli of studied samples during storage time of (0–50) days measured at 1 Hz. Table S5: Values of viscous moduli of studied samples during storage time of (0–50) days, measured at 1 Hz. Table S6: Values of phase shift of studied samples during storage time of (0–50) days.
