# Supplementary material for: The Composition Optimization of Curcumin-Loaded Double Oil–Water–Oil Emulsions and Their Stability Evaluation
Source: Molecules. 2024 Aug 26;29(17):4035. doi: 10.3390/molecules29174035 (PMC11396728; doi:10.3390/molecules29174035)
Supplement: Supplementary file 1 [file molecules-29-04035-s001.zip › Appendix S3 Rheology.pdf]

Appendix S3: Results of rheological measurements.

Table S4. Values of elastic modulus for samples during storage time of (0-50) days, measured at 1 Hz.

Sample	G'(Pa)			
	Day 0	Day 15	Day 30	Day 50
MEO	52.8±2.1	51.4±1.8	57.1±1.5	58.8±1.4
MES	41.8±1.7	43.5±2.1	49.2±1.3	48.2±1.2
MEP	38.1±0.49	43.1±1.6	49.7±1.5	52.9±0.7
MEF	58.8±1.4	59.4±1.5	60.5±1.7	66.1±0.9
MEK	1301666.6±1250	1567333.3±998	1543000.0±1100	1599666.6±1203

Table S5. Values of viscous modulus for samples during storage time of (0-50) days, measured at 1 Hz.

Sample	G" (Pa)			
	Day 0	Day 15	Day 30	Day 50
MEO	20.6±1.2	20.9±0.7	22.1±0.5	23.9±0.8
MES	12.2±1.1	15.5±0.6	20.6±0.5	24.2±1.2
MEP	22.09±0.5	23.9±0.9	32.32±1.1	32.6±0.6
MEF	21.9±1.3	23.9±0.7	24.4±0.6	24.6±0.37
MEK	202833.3±658	164066.7±542	165100±399	179200±601

Table S6. Values of phase shift for samples during storage time of (0-50) days.

Sample	δ (°)			
	Day 0	Day 15	Day 30	Day 50
MEO	20.03±0.6	20.7±0.4	22.1±0.7	22.9±0.9
MES	16.8±0.5	19.6±0.3	20.5±08	23.2±0.4
MEP	19.7±0.5	20.2±06	24.0±1.0	24.5±0.4
MEF	18.4±0.5	23.4±0.6	24.09±1.1	24.5±0.3
MEK	6.0±0.3	8.1±0.1	9.5±0.2	12.5±0.3
