# Supplementary material for: The Composition Optimization of Curcumin-Loaded Double Oil–Water–Oil Emulsions and Their Stability Evaluation
Source: Molecules. 2024 Aug 26;29(17):4035. doi: 10.3390/molecules29174035 (PMC11396728; doi:10.3390/molecules29174035)
Supplement: Supplementary file 1 [file molecules-29-04035-s001.zip › Supplementary Materials_OWO.pdf]

Table S1: Optimization method for the inner phase of emulsion.

Run	Factor X_1<br>Oil<br>(ml)	Factor X_2<br>Temperature<br>(°C)	Factor X_3<br>Homogenization<br>(RPM) [a]	Response 1<br>CPI30 [b]	Response 2<br>ESI [c]<br>(%)
1	17.5 (0)	47.5 (0)	20000 (0)	25	27.3±0.4
2	5 (-1)	25 (-1)	15000 (-1)	90	91.0±1.4
3	17.5 (0)	47.5 (0)	20000 (0)	25	27.4±0.2
4	17.5 (0)	15 ($-\alpha$)	20000 (0)	95	89.0±1.4
5	5 (-1)	25 (-1)	25000 (+1)	100	99.0±1.4
6	30 (+1)	70 (+1)	15000 (-1)	10	0.0±0.0
7	17.5 (0)	47.5 (0)	20000 (0)	25	25.8±1.1
8	17.5 (0)	47.5 (0)	20000 (0)	25	29.3±1.1
9	17.5 (0)	47.5 (0)	20000 (0)	25	27.3±0.4
10	17.5 (0)	47.5 (0)	20000 (0)	25	27.4±0.2
11	30 (+1)	25 (-1)	15000 (-1)	50	89.8±0.4
12	17.5 (0)	47.5 (0)	28000 ($+\alpha$)	32	34.5±0.7
13	2 ($-\alpha$)	47.5 (0)	20000 (0)	50	35.0±0.3
14	30 (+1)	70 (+1)	25000 (+1)	0	0.0±0.0
15	30 (+1)	25 (-1)	25000 (+1)	95	95.9±0.1
16	17.5 (0)	47.5 (0)	11591 ($-\alpha$)	31	20.0±0.7
17	5 (-1)	70 (+1)	15000 (-1)	5	9.6±0.5
18	17.5 (0)	85.3 ($+\alpha$)	20000 (0)	0	0.0±0.0
19	5 (-1)	70 (+1)	25000 (-1)	8	5.1±0.1
20	38 ($+\alpha$)	47.5 (0)	20000 (0)	28	21.5±0.7

[a] RPM - rotations per minute. [b] CPI_{30} - creaming index after 30 days of storage. [c] ESI - emulsion stability index.

Table S2: Optimization method for multiple emulsions.

Run	Factor X_1 Oil (ml)	Factor X_2 Emulsifier (ml)	Factor X_3 Homogenization (RPM) [a]	Response 1 CPI30 [b]	Response 2 ESI [c] (%)
1	50 (0)	0.55 (0)	6500 (0)	95	90.3±0.4
2	70 (+1)	0.1 (-1)	12000 (+1)	40	45.2±0.1
3	30 (-1)	0.1 (-1)	12000 (+1)	0	45.4±0.2
4	30 (-1)	1 (+1)	1000 (-1)	95	49.8±0.4
5	70 (+1)	0.1 (-1)	1000 (-1)	90	14.8±0.4
6	50 (0)	0.55 (0)	6500 (0)	90	95.3±0.4
7	50 (0)	0.55 (0)	6500 (0)	100	95.5±0.7
8	50 (0)	0.55 (0)	6500 (0)	90	95.2±0.5
9	50 (0)	0.55 (0)	15 000 (+α)	85	100.0±0.0
10	50 (0)	0.55 (0)	0 (-α)	85	0.0±0.0
11	30 (-1)	1 (+1)	12000	100	99.9±0.1
12	70 (+1)	1 (+1)	1000 (-1)	15	50.6±0.8
13	70 (+1)	1 (+1)	12000 (+1)	0	99.3±1.1
14	16 (-α)	0.55 (0)	6500 (0)	30	95.3±0.4
15	30 (-1)	0.1 (-1)	1000 (-1)	15	15.3±0.4
16	85 (+α)	0.55 (0)	6500 (0)	100	80.8±1.1
17	50 (0)	1.3 (+α)	6500 (0)	95	99.0±1.4
18	50 (0)	0 (-α)	6500 (0)	90	0.0±0.0
19	50 (0)	0.55 (0)	6500 (0)	95	90.5±0.7
20	50 (0)	0.55 (0)	6500 (0)	95	90.8±0.4

[a] RPM - rotations per minute. [b] CPI_{30} - creaming index after 30 days of storage. [c] ESI - emulsion stability index.

Table S3: Results of encapsulation efficiency of multiple emulsions during the storage period.

	EE (%) [1]			
Sample	0 day	15 day	30 day	50 day
DEO [2]	92.29±0.40^{Aa}	88.14±0.43^{Ab}	81.51±2.06^{Ab}	75.44±1.21^{Ac}
DES [3]	89.32±2.10^{Aa}	82.35±1.09^{Aa}	71.92±2.39^{Aa}	63.88±2.72^{Aa}
DEF [4]	90.80±3.35^{Aa}	84.31±1.49^{Aa}	75.36±1.20^{Aa}	70.43±0.43^{Ab}
DEP [5]	94.08±0.58^{Aa}	86.85±0.86^{Ab}	79.46±0.98^{Ac}	72.54±0.30^{Ad}
DEK [6]	93.79±1.03^{Aa}	93.58±0.78^{Ba}	92.69±0.67^{Ba}	90.96±0.82^{Bb}

[1] EE - encapsulation efficiency. [2] DEO - double emulsion with olive oil. [3] DES - double emulsion with sunflower oil. [4] DEF - double emulsion with flaxseed oil. [5] DEP - double emulsion with pumpkin oil. [6] DEK - double emulsion with coconut oil.

[a] Means within a row (the difference between stored time of one sample); comparing the same followed by different lowercase superscript letters differ significantly ($P < 0.05$). [A] Means within a column (the difference between the samples); comparing the same followed by different superscript letters differ significantly ($P < 0.05$).

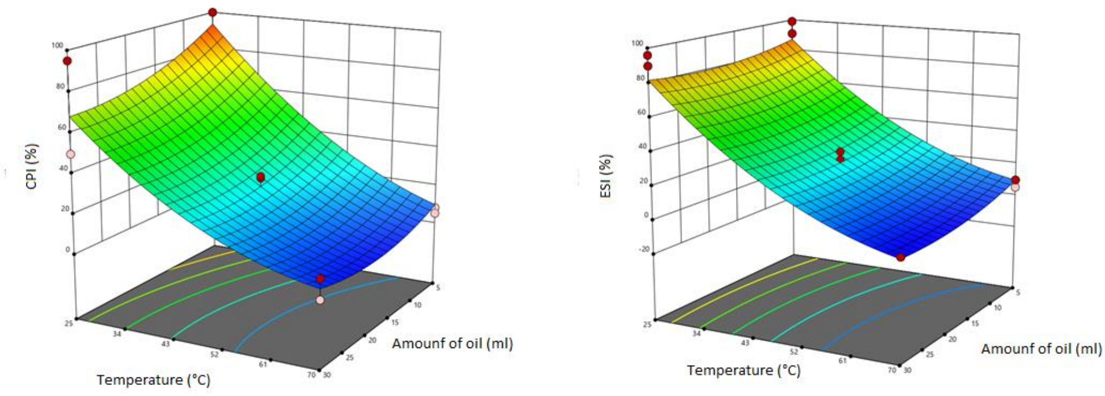

Figure S1: Response surface plots showing the effect of preparation temperature, oil amount, and homogenization method on creaming index CPI (%) and emulsion stability index ESI (%) for multiple emulsion optimization.

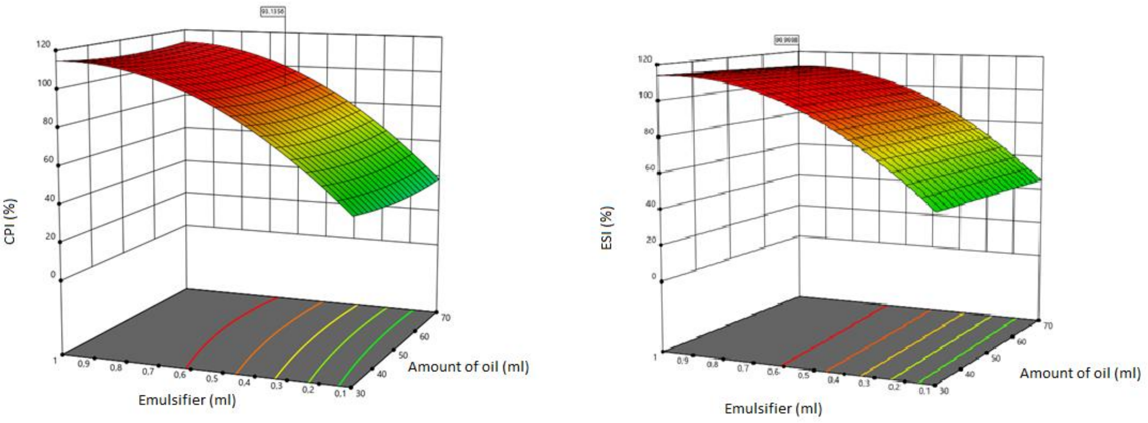

Figure S2: Response surface plots showing the effect of emulsifier amount, oil amount, and homogenization method on creaming index CPI (%) and emulsion stability index ESI (%) for multiple emulsion optimization.
